# Schwerpunkt: 20 Jahre Euro

**DOI:** 10.1007/s41358-022-00331-5

**Published:** 2022-09-28

**Authors:** Karsten Schäfer

**Affiliations:** Institut für Sozialwissenschaften, Abteilung Politikwissenschaft, Universität Koblenz-Landau, Landau, Deutschland Kaufhausgasse 9, 76829

Jubiläen bieten gemeinhin die Möglichkeit, Bilanz zu ziehen. Insoweit ist es keine Überraschung, dass die Gratulantinnen und Gratulanten aus den Reihen der EU-Institutionen, die den Euro in diesem Jahr zu seinem zwanzigjährigen Bestehen^1^ beglückwünschen, nahezu einhellig seine politisch-historisch sowie ökonomisch bedeutsame Entwicklung für Europa und die gesamte Welt betonen: „Der Euro ist nicht nur eine der stärksten Währungen der Welt. Er ist in erster Linie ein Symbol für die europäische Einheit“, erklärte EU-Kommissionspräsidentin Ursula von der Leyen. Und Paschal Donohoe, Präsident der Euro-Gruppe, sagte: „Der Euro hat sich im Zuge der Bewältigung großer wirtschaftlicher Herausforderungen bewährt. So hat unsere Reaktion auf die COVID-19-Pandemie gezeigt, dass wir Länder des Euro-Währungsgebiets gemeinsam mehr erreichen können als jedes für sich allein“ (Europäische Kommission 2022a).

Tatsächlich lassen sich derartige Einschätzungen durch den Blick auf ökonomische Kennzahlen untermauern. Insbesondere wird erkennbar, dass sich der Euro – anders als vor seiner Einführung vielerorts prognostiziert wurde^2^ – in Sachen Preisentwicklung in den vergangenen zwei Jahrzehnten als überaus stabil erwiesen hat (vgl. hierzu beispielsweise Bundesministerium der Finanzen 2022). Während der Krisen der vergangenen Jahre – vor allem in der Eurokrise sowie während der COVID-19-Pandemie – hat er sich als wichtiger „Stabilitätsanker“ erwiesen, ohne den der europäische Binnenmarkt nicht funktionieren würde (Tokarski 2022). Gegenwärtig nutzen mehr als 340 Mio. Menschen in 19 EU-Staaten den Euro als Zahlungsmittel, der sich nach dem US-Dollar als zweitwichtigste Währung weltweit etabliert hat (Europäische Kommission 2022a).

Auch außerhalb des Euroraums hat die Währung deshalb zwischenzeitlich eine große Strahlkraft entwickelt. Das verdeutlicht der Blick auf die Staaten, in denen der Euro (noch) nicht die offizielle Landeswährung ist. Laut einer aktuellen Eurobarometer-Befragung befürworten rund 60 % der Befragten^3^ die Einführung des Euro in ihrem Land (Europäische Kommission 2022c). Heterogener, jedoch insgesamt ebenfalls positiv gestalten sich die Zustimmungswerte innerhalb des Euroraums. Die Daten zeigen, dass 63 % der Befragten aus den EU-Ländern, deren Währung der Euro ist, seine Auswirkungen auf die wirtschaftliche Performanz der EU in den vergangenen 20 Jahren als „positiv“ einstufen (Europäische Kommission 2022b, S. 22). Vor diesem Hintergrund fällt auch die wissenschaftliche Auseinandersetzung mit der Entwicklung des Euro im Ergebnis größtenteils positiv aus. Wenngleich unstrittig ist, dass die Reformen der letzten Jahre – insbesondere die Schaffung des Fiskalpakts, der Europäischen Bankenunion sowie des Europäischen Stabilitätsmechanismus (Fratzscher et al. 2018) – nicht alle strukturellen Probleme beheben konnten und die aktuellen ökonomischen und (geo-)politischen Herausforderungen den Euro zusätzlich belasten, lässt sich festhalten, dass dieser unter dem Strich als ökonomischer wie politischer Erfolg angesehen werden kann.

Der vorliegende Focus greift verschiedene Facetten der wissenschaftlichen Auseinandersetzung mit der europäischen Währung auf. Er beleuchtet die ersten 20 Jahre Euro mit drei Beiträgen aus unterschiedlichen Perspektiven: Berthold Busch und Jürgen Matthes fordern Solidität in allen Mitgliedsstaaten des Euroraums – diese müsse nun zwingend auf die Solidarität folgen, die einzelne Euro-Länder durch die Europäische Union in den vergangenen Jahren und insbesondere in Krisenzeiten erfahren haben, argumentieren die Autoren. Roland Sturm skizziert in seinem Beitrag das wechselvolle und bisweilen schwierige Verhältnis Großbritanniens zum Euro. Dieser war lange der „elephant in the room“, wenn es um die Beziehung Großbritanniens zur EU ging, ehe der Brexit das Thema (vorerst) beendete, schildert Sturm. Der Beitrag von Benjamin Braun, Donato di Carlo und Sebastian Diessner thematisiert abschließend die von der Europäischen Zentralbank (EZB) stetig vorangetriebenen Bemühungen um strukturelle Reformen innerhalb der EU, jenseits ihres eigentlichen Mandats. Die Autoren rekurrieren bei ihrer Analyse auf den theoretischen Rahmen, den der Wirtschaftstheoretiker Karl Polanyi in seinem Werk „The Great Transformation“ entwickelt hat, um auf diese Weise das strukturreformfreudige Verhalten der EZB in der jüngeren Vergangenheit nachvollziehbar zu machen.

## Footnotes

1 Zum 01.01.2002 wurde der Euro in damals zwölf Mitgliedsstaaten der Europäischen Währungsunion als Bargeld eingeführt. Als Buchgeld wurde er bereits ab dem 01.01.1999 genutzt.

2 Zum Beispiel war in Deutschland vor der Euro-Einführung hinsichtlich befürchteter Preissteigerungen der Begriff des „Teuro“ zeitweise sehr präsent.

3 Befragt wurden Personen in den Staaten Bulgarien, Kroatien, Tschechien, Ungarn, Polen, Rumänien und Schweden. Die größte Zustimmung zur Einführung des Euro zeigt sich mit 77 % in Rumänien.
